# Exploring Effective Sensory Experience in the Environmental Design of Sustainable Cafés

**DOI:** 10.3390/ijerph17238957

**Published:** 2020-12-02

**Authors:** Yen-Cheng Chen, Hsiang-Chun Lin

**Affiliations:** 1 Department of Applied Science of Living, Chinese Culture University, Taipei City 111, Taiwan; cyc4@g.pccu.edu.tw; 2 Department of Hotel Management, Jin Wen University of Science & Technology, New Taipei City 231, Taiwan; 3 Department of Geography, National Taiwan Normal University, Taipei City 106, Taiwan

**Keywords:** café, green ambience, Delphi method, indicator design

## Abstract

The aim of this study was to explore and construct spatial indicators suitable for green café ambience. The indicators were further empirically verified. A three-round questionnaire survey, based on the Delphi method, was conducted with 15 experts, including university professors (food and beverage services management and interior environmental design), café operators, and personnel from government agencies. Data were collected, and the results on the characteristics of the repeated feedback from the experts were convergent. Thirty-six indicators suitable for the design of green café ambience were extracted, of which 17 were verified by actual cafés as highly operable. The five-sense indicators of sustainable green ambience design obtained in this study can facilitate positive customer experiences and enhance the appeal of maintaining sustainable green trends for cafés. These indicators can also provide references for café operators in business planning and green café ambience design.

## 1. Introduction

In recent years, the issue of green sustainability has been attributed increasingly more importance around the world, and the concept of green consumption is trending. The question of how to apply this concept to the food and beverage service industry to reduce its adverse environmental impact is becoming important in the catering industry [1,2,3]. According to Chiang [4], in Taiwan, cafés have flourished in the streets and alleys of cities in recent years, providing quality dining spaces, food service, and situational experiences and becoming one of the fast-growing food and beverage industry sectors as well as an important part of the social life of locals. Consumers go to cafés not just to drink coffee but also to enjoy their ambience [5].

When consumers choose a restaurant, environmental factors affect their choice [1,3,6]. Influenced by the awareness of green sustainability, consumers have realized that green spaces function as ecological environments, and the introduction of a green theme can attract customers and thus create economic value [7]. Previous studies have also pointed out that café operators need to react quickly to new trends in coffee consumption and be able to provide new experiences to consumers [8]. Usually, the creation of an appropriate environment and ambience can benefit the sale of food and beverage products, provide positive sensory stimuli to consumers, and produce a unique style for the store that attracts consumers and strengthens their loyalty [9,10,11]. According to Kim and Jang [12], café managers need to foster a relationship between consumers and such venues so that consumers form a sense of place attachment. However, in the design of cafés in Taipei, Taiwan, in the past, only the visual sense has been paid attention to, while consumers’ overall comprehensive feeling based on the information they receive through their 5 senses (touch, sight, hearing, smell, and taste) and then assess this information via the brain has been overlooked. Therefore, the innovation and contribution of this study stem from its evaluation of green sustainability-themed ambience indicators in 5 dimensions, which supports the more comprehensive design of the necessary ambience for sustainable green cafés.

Since the Industrial Revolution in the eighteenth century, industrialization has caused serious environmental damage, and environmental protection and sustainable management have become global issues. To maintain economic growth while mitigating environmental damage, many governments have been actively promoting sustainable green industries, to which the hospitality and food services industries belong [1,3]. Xu and Jeong [13] found that most environmental protection actions in the past have focused on environmental problems caused by the manufacturing industry and that, more recently, as consumer environmental awareness has improved, people have gradually realized that the hospitality and tourism industries also exert an impact on the environment. The food and beverage services industry is an important part of the tourism industry, consuming a significant proportion of resources used by the tourism system as a whole [2]. However, while the issue of green sustainability has begun to receive attention in the food and beverage industry, sustainable environmental design for cafés in Taiwan has rarely been addressed in depth, indicating a research gap. Thus, in this study, we wanted to answer the following questions: (1) What are the important indicators in the analysis of options for the ambience design of sustainable café in Taiwan? (2) What are indicators that can be used in analysing options that can be implemented in cafés?

Therefore, in this study, based on the basic principles of the Green Restaurant Association (GRA), we examined the development of green restaurants and the potential development patterns of green cafés and their integration of different aspects of ambience; based on the 5 dimensions of ambience proposed in a previous study [14], we investigated and constructed 5 system dimensions, i.e., auditory ambience, visual ambience, tactile ambience, olfactory ambience, and taste ambience, according to selection criteria with respect to balance, feasibility, operability, independence, and systematicity to construct an index system for the evaluation of green café ambience design that is of practical significance to the coffee and beverage services industry and consumers. Therefore, the objectives of this study were as follows:

1. To investigate and construct the indicators that are important to and suitable for green café ambience design
2. To empirically analyse indicators with high operability that are suitable for green café ambience design.

## 2. Literature Review

### 2.1. Green Food and Beverage Industry

In the late 1990s, due to environmental pollution, countries began to attach importance to environmental protection and sustainable development [3]. Compared with the concept of general environmental protection, green environmental protection started late in the catering industry, but because the catering industry is prone to a plethora of resource waste and pollution issues, the concept of “green restaurants” emerged in response to the call for green environmental protection [15,16].

The term “green” has become a synonym for pollution abolishment and environment protection, signifying health and sustainability [3]. The essence of green restaurants is to protect the global environment and create an environmentally sustainable catering industry in a way that reduces adverse impacts on the environment while providing products and services to meet people’s needs and improve their quality of life [2]. The international environmental protection movement that has developed since the 1970s has re-examined the environmental damage caused by rapid economic development after the Industrial Revolution and has prompted people to pay increasingly more attention to the idea of environmental protection. The state, enterprises, and citizens can contribute to the sustainable development of the environment, and “eating and drinking”, the most common behaviour in daily life, could be the most feasible way to promote the concept of a green diet [1,15].

The GRA was established in 1990; their mission is to help the catering industry become an environmentally sustainable business while promoting energy conservation. An increasing number of restaurants are designing suitable plans for environmental protection measures. For example, food material procurement, tableware material, recycled paper use, waste processing, water resource use, etc., have become important aspects in the design of green restaurants. In 1995, the GRA issued a green restaurant standard that includes 7 items, i.e., saving water, reducing waste, using sustainable furniture and building materials, using sustainable food materials, saving energy, reducing the use of disposable products, and reducing the use of chemical products and pollutants, and they advocated for the annual education of employees regarding environmental protection [17].

### 2.2. Ambience

With economic growth and social structural transformation, people’s requirements for quality of life are increasing day by day, which is reflected, in particular, in the transition from dining solely for the purpose of having a meal to dining with a focus on the food itself and the restaurant environment, which are aspects that are becoming increasingly emphasized [18]. Baker et al. [19] suggest that consumers’ sentiment is often related to consumption ambience; the design and creation of ambience can create a favourable consumption situation for an establishment, fostering positive consumer emotions [20]. Liu and Jang [21] also note the significant impact of ambience on customer emotions. Clearly, consumer demand for catering quality no longer concerns only dining but also concerns eating well, so the physical environment of the restaurant and dining ambience affect their consumption mood, which is closely associated with ambience. Schmitt [14] argue that the creation of ambience can affect consumers’ perceptions of products. In a literature review, Turley and Milliman [22] note that environmental factors such as lighting, music, temperature, smell, interior design, and tableware placement are important for creating ambience in a restaurant. Therefore, providing a good environment can enhance consumers’ consumption experience, making them happy.

When cafés flourish, this indicates that the consumer population is also expanding. Current consumers have developed different coffee consumption patterns from those of past consumers. Previously, people went to cafés for social, leisure, and dining purposes, but now, they want to relax and relieve stress at cafés [23,24]. With this transformation, when choosing a café, consumers pay attention not just to internal factors, such as products and services, but also to external factors, such as the ambience of the café itself [25]. The experience created by ambience in a café and the design and creation of ambience have become the main factors that attract consumers and influence their consumption behaviours [26]. Schmitt [14] note that the development of sentiment is most intense during consumption, for which the consumption ambience is the most important; consumers construct their perceptions of the world through the 5 senses, i.e., touch, sight, hearing, smell, and taste, which enable customers to have good consumption experiences, deepening their positive impressions while increasing their return rate. Therefore, multi-sense designs have become a trend for stores to increase consumer appeal [9,15,27,28].

### 2.3. Ambience Creation and Sensory Experience

Chen et al. [6] show that proper ambience creation and sensory experience have positive impacts on consumer emotions. The design of a restaurant’s environment and ambience can enable consumers to generate more consumption desires and specific attitudes and behavioural responses [29]; thus, restaurant managers should improve service quality and adopt marketing strategies that meet the needs of the restaurant to improve consumer satisfaction [30]. Turley and Milliman [22] also indicate that a restaurant’s ambience can positively affect consumer satisfaction with meals.

Furthermore, Leena [31] argue that in addition to providing meals, to increase competitiveness, the catering industry needs to provide consumers with a comfortable dining environment. Importantly, the design of a restaurant’s ambience affects consumer satisfaction and behaviour [32]. To improve consumer satisfaction, food and beverage service providers must understand and design the appropriate ambience for their establishments to improve consumers’ positive emotions, which in turn will affect an establishment’s market competitiveness. Moreover, Rhee et al. [29] show that the design of a restaurant’s ambience enables consumers to have higher consumer sentiment and specific attitudes and behavioural responses. Hamlin [33] also note that sensory designs have great impacts on consumers and that emotions can play a key role for many consumers. Consumers are paying an increasing amount of attention to an establishment’s ambience when they make patronage choices because the ambience affects their emotions [34].

Therefore, sensory stimulation-focused ambience designs can trigger consumers’ consumption sentiment, enhance their purchase intention, and lengthen their time spent in an establishment; ambience design for different consumer groups can have a key impact, the degree of which should not be underestimated [23,35]. In summary, environmental issues have become the focus of attention in various countries. If we can introduce the concept of green restaurants to Taiwan, it will be feasible to apply this concept in cafés through the creation of a five-sense ambience experience so that consumers’ green and sustainable ambience experience is taken into account in the design of green and environmentally friendly cafés and so that their consumption experience and overall image of the café are enhanced

## 3. Materials and Methods

Based on the Delphi method, we collected and analysed data, determined the indicators, and constructed a research framework according to the aforementioned research motivation, objectives, and literature review; the details of those processes are described below.

### 3.1. Indicator Selection

In this research, we drew on the green restaurant standard proposed by the GRA; the five-sense experience proposed by Schmitt [14], Bitner [36] and Kotler [37]; and the guidance on choice criteria that take into account balance, feasibility, operability, independence and systematic aspects recommended by Dalkey and Helmer [38] on the basis of the 5 dimensions of ambience. Based on these references, we constructed 5 system dimensions: auditory ambience, visual ambience, tactile ambience, olfactory ambience, and taste ambience.

### 3.2. Selection of Subjects for the Delphi Method-Based Questionnaire Survey

This aim of this study was to establish criteria for the design of green ambience in cafés. Because the scope of issues is associated with the management of food and beverage services and the planning of environmental policy and infrastructure, experts from these fields were included as subjects. According to Dalkey and Helmer [38], for the Delphi method, a sample size of over 10 individuals can decrease the group-level error and thus achieve the highest reliability, which can be achieved by including 51–0 individuals in the case of a heterogeneous group. The participants of this study included college professors (catering services management and interior design), experts from government agencies responsible for overseeing catering businesses and protecting the environment, and café operators, constituting a heterogeneous group. For the rigour of the study, the number of experts recruited for this study was set at 15 (Table 1).

### 3.3. Questionnaire Design and Distribution

Green et al. [39] demonstrated that for the Delphi method, 2 or 3 questionnaire rounds will lead to consensus, and therefore, a further increase in the number of questionnaire rounds will not affect the results because respondents will no longer change their answers to the questionnaire items. Therefore, in this study, we conducted a three-round questionnaire survey to reach a consensus among the participating experts. The questionnaire was scored using a Likert scale (very important or very easy = 5 points; important = 4 points; fair = 3 points; not so important = 2 points; not important at all = 1 point; and no opinion or cannot decide = 0 points).

After the first-round questionnaire was collected, the average score and standard deviation of each candidate indicator were calculated. For the second-round questionnaires, the scores for each candidate indicator and the average score for each candidate indicator obtained in the first-round questionnaire were included; this allowed the experts could score the indicators in the second-round questionnaire in terms of their importance and operability in reference to the answers that the others experts provided and the average score for each indicator in the previous round.

**Figure 1:**
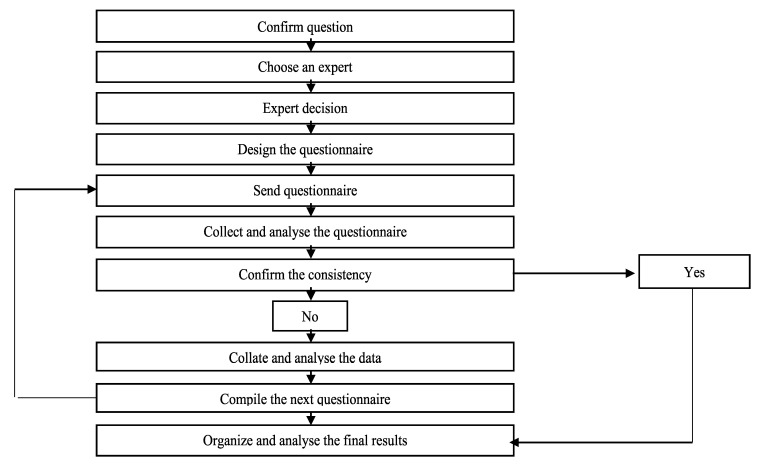
Delphi process.

Further, as described above, based on the experts’ suggestions and comments made in the first round, which were included in the second-round questionnaire, the wording of some candidate indicators was revised without changing the original meaning, and 6 new candidate indicators were added. This process resulted in the second-round questionnaire, which contained a total of 103 candidate indicators. After the second-round questionnaire was collected, the average score of the experts for each candidate indicator was calculated, which, together with the score for each candidate indicator provided by each expert, was included in the third-round questionnaire to provide information for scoring the indicators in terms of their importance and operability. This process resulted in the third-round questionnaire. Finally, after the third-round survey was completed using the formal questionnaire, stability analysis was performed on the first-round and the second-round questionnaires and the second-round and third-round questionnaires. The average score and standard deviation of each candidate indicator in the third-round questionnaire were calculated (Figure 1).

### 3.4. Choice and Verification of Actual Cafés and the Design of the Verification Questionnaire for Actual Cafés

To verify the indicators of green café ambience designed in this study, after applying the Delphi method, we issued questionnaires to 10 non-chain cafés in Taipei to empirically strengthen the applicability of the findings from this study. Cafés that had more than 20 tables, sold coffee drinks and light food, were located in business areas of Taipei City, and had a unique decoration style were chosen. A total of 36 indicators of green café ambience design with high operability were analysed and verified through a questionnaire survey administered to on-site to owners or managers of 6 cafés; the questionnaires were answered anonymously and were scored using a Likert scale (very important or very easy = 5 points; important = 4 points; fair = 3 points; not so important = 2 points; not important at all = 1 point; and no opinion or cannot decide = 0 points).

### 3.5. Data Processing and Analysis

Based on the questionnaires issued in this study, in addition to the collation and revisions based on experts’ opinions, the data for the quantitative part of the questionnaires were measured using the average score as the standard measure of the importance of the indicators. According to Linstone and Turoff [40], stability can be used to determine whether experts’ opinions on questionnaire items are consistent and stable, the details of which are described below.

#### 3.5.1. Stability Analysis

In the stability analysis, after the administration of the first-round and second-round questionnaires and the second-round and third-round questionnaires, the numbers of respondents who revised their scores for each indicator (scored based on a 5-point Likert scale, in which very important = 5 points and not important at all = 1 point) were added and then divided by 2, yielding the number of respondents who revised their opinions. This number was then divided by the number of total respondents who participated in the questionnaire survey, yielding the stability index.

#### 3.5.2. Screening of Averages

Through the feedback component of the Delphi method, each item of the recovered questionnaire was listed, and its importance and operability were determined; furthermore, the score provided by each participating expert was provided so that others’ feedback on each item could be known without the need for the experts to directly communicate with each other and so that the feedback could be used as a reference [38].

After the experts’ opinions for the first-round questionnaire were integrated, the average score of each candidate indicator was calculated; then the average of each indicator for the first-round questionnaire was included in the second-round questionnaire to provide a reference for the experts when they answered the second-round questionnaire; after the second-round questionnaires were recovered, the average of each indicator was also calculated individually and included in the third-round questionnaire, and the experts’ opinions were similarly revised for the third-round questionnaire. The above processes were repeated until the questionnaire results were consistently convergent when statistical analysis of the results was performed. In addition, after the third-round questionnaire survey was completed, indicators with a stability index greater than 15% were excluded through the stability analysis.

Finally, based on the overall average score, the average score for each dimension, and the importance score (of 4 points or above) obtained from the third-round questionnaire, the average selection process was performed on each of the retained indicators. Through the observation of the structure and distribution of all averages, a suitable index was chosen as the basis for the third-round screening of the indicators. The screening principle used to generate this value needed to allow a complete structure of the overall index system for green café ambience to be maintained, i.e., with at least one indicator from each dimension with a final score of 4 points or above retained in the final list of indicators based on the average importance score of the third-round questionnaire indicators (with a score of 4 or above).

#### 3.5.3. Discussion based on the Standard Deviation

The consistency of the experts’ opinions was measured using the standard deviation; the greater the standard deviation was, the higher the inconsistency on the item among the experts, and vice versa [38].

The calculation of the standard deviation was intended to examine the difference in experts’ views regarding each questionnaire item. An indicator with a high and significant standard deviation value in the first-round questionnaire suggested that the experts’ views on this item were still highly inconsistent, and vice versa. The calculation was performed until the third-round questionnaires were collected, and if the standard deviation value of a certain indicator was still significant, the indicator was further discussed.

## 4. Results

### 4.1. Questionnaire Distribution and Recovery

Questionnaires were distributed to experts, including scholars (food and beverage services management or environmental management), personnel from government agencies and café operators, by mail or e-mail or by on-site distribution. The three-round questionnaire survey was completed in approximately 2 months. A total of 15 questionnaires were recovered from the participants who completed the entire survey process, for a recovery rate of 100%.

### 4.2. Analysis of Importance Indicators

Stability analysis is mainly used to determine whether the scores of an indicator are convergent. In this study, the calculation formula for stability proposed by Linstone and Turoff [40] was used to calculate the stability index. After the third-round questionnaires were recovered, stability analysis was performed for each indicator by first measuring the stability of the score of each indicator provided by each expert in the first-round questionnaire and that in the second-round questionnaire and then performing the same stability measurement on the scores of each indicator provided by each expert in the second-round and the third-round questionnaires once the third-round questionnaires were collected. A stability index value below 15% suggested that the experts’ opinions on this indicator reached a stable level and that the indicator could be included in the index system. The stability analysis results suggested that 20 indicators did not meet the stability index standard, i.e., had index values lower than 15%:

(1) 2 indicators in the auditory ambience dimension, i.e., “It is appropriate for green cafés to play audio recordings of the sound of rain” and “It is appropriate for green cafés to play violin music”;
(2) 6 indicators in the visual ambience dimension, i.e., “It is appropriate for green cafés to use plastic as the main material for tables and chairs”, “It is appropriate for green cafés to use candlelight”, “It is appropriate for green cafés to use neon lights”, “It is appropriate for green cafés to use ceiling lamps”, “It is appropriate for green cafés to use ceiling fans”, and “It is appropriate for green cafés to have their service staff dressed predominantly in green”;
(3) 6 indicators in the tactile ambience dimension, i.e., “It is appropriate for green cafés to use dehumidifiers to keep the air dry”, “It is appropriate for green cafés to use plastic utensils such as trays, cups, spoons, knives, forks, etc.”, “It is appropriate for green cafés to use a plastic floor”, “It is appropriate for green cafés to use plastic chairs”, “It is appropriate for green cafés to use steel chairs”, and “It is appropriate for green cafés to use plastic tables”;
(4) 2 indicators in the olfactory ambience dimension, i.e., “It is appropriate for green cafés to have a food aroma”, and “It is appropriate for green cafés to use incense”; and
(5) 4 indicators in the taste ambience dimension, i.e., “It is appropriate for green cafés to predominantly provide meat-based food”, “It is appropriate for green cafés to provide fried food”, “It is appropriate for green cafés to provide cured food”, and “It is appropriate for green cafés to provide grilled food”.

The above indicators did not meet the stability analysis criterion, meaning that experts did not reach a consensus on each of these indicators; therefore, these indicators were excluded, while the remaining candidate indicators had stability index values lower than 15%. After the above 20 candidate indicators were excluded, the remaining 83 candidate indicators were retained for the subsequent average screening stage.

The following criterion was used for the average screening: with the purpose of not disrupting the system structure, an appropriate average was chosen as the standard for the removal of candidate indicators. In this study, the candidate indicators were screened based on 3 criteria: (1) the overall average score (3.5 points); (2) the average score for each dimension (after calculation, the average scores for the dimensions were as follows: audible ambience, 3.7 points; visual ambience, 3.5 points; tactile ambience, 3.6 points; olfactory ambience, 3.2 points; and taste ambience, 3.4 points); and (3) the importance score (greater than 4 points).

The results are shown in Table 2. Ultimately, to ensure the integrity of the overall system structure and the high scores of the selected indicators, an importance score above 4 points for each dimension scored in the third-round questionnaire was used as the screening criterion for this stage.

Therefore, we used this criterion as the basis for selecting indicators, and the system not only included indicators with high importance scores (4 points or above) but also excluded those with a stability index below 15%; however, the average importance score was lower when the indicators for all dimensions were considered. Finally, after the candidate indicators with a stability index value above 15% and an average score below 4 points were deleted, a total of 36 candidate indicators were selected.

**Figure 2:**
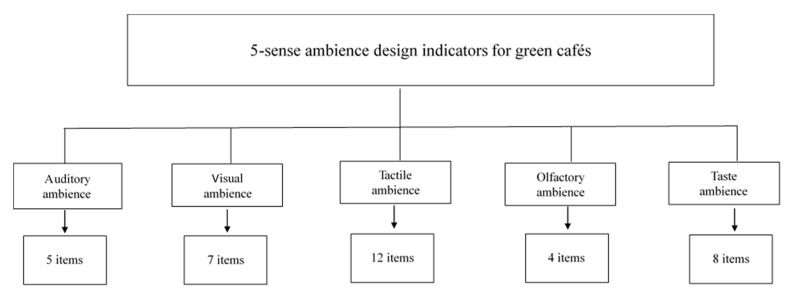
Indicators for green café ambience design.

The establishment of the above importance indicators was achieved through the following processes: after repeated feedback from the three-round expert questionnaire surveys, initial indicators with stability index values below 15% on the second- and third-round questionnaires for which the experts reached a consensus were chosen and subsequently screened based on the average for each dimension in the third-round questionnaire. Ultimately, a total of 36 indicators were selected as indicators of green café ambience: 5 auditory ambience indicators, 7 visual ambience indicators, 12 tactile ambience indicators, 4 olfactory ambience indicators, and 8 taste ambience indicators (Figure 2).

### 4.3. Analysis of the Operability of the Indicators

In this study, the average score in the third-round questionnaire (easy, 4 points) was used as the criterion to measure the operability of the selected 36 indicators; i.e., indicators with an average value of 4 points or above were considered highly operable, as they had an operativity level of “easy” or above (Table 3).

After the experts’ three-round analyses of the indicators, we chose 10 cafés to verify the selected indicators. The managers of the front and back lines and staff were asked to evaluate the indicators for the design of green café ambience; the evaluations were scored using a 5-point Likert scale, and the average scores were compared with the selected indicators to verify that these indicators could truly serve as the indicators for the design of green café ambience.

Last, based on the average scores of the 36 selected indicators, the average scores derived from the verification by actual café managers, and the criterion of an average value of 4 points or higher and small variation, 17 indicators with operability levels of “easy” or above that were scored in the verification were ascertained (Table 4).

## 5. Discussion

### 5.1. Selected Indicators

In terms of the average operability score, 3 items, i.e., “It is appropriate to plant flowers and trees outside green cafés” in the visual ambience dimension (4.93 points), “It is appropriate for green cafés to have a coffee aroma” in the olfactory ambience dimension (4.83 points), and “It is appropriate for green cafés to provide predominantly fresh vegetables as food” in the taste ambience dimension (4.76 points), had high scores, indicating that the experts agreed that the 3 indicators are highly important for the design of green café ambience and are highly feasible.

In terms of the overall dimensions, auditory ambience (3.7 points), tactile ambience (3.6 points), and visual ambience (3.5 points) all had an average importance score above 3.5 points, indicating that these 3 dimensions are important indicators of green café ambience design.

### 5.2. Empirical Verification

#### 5.2.1. Auditory Ambience Dimension

In this dimension, the item “It is appropriate for green cafés to play audio recordings of the sound of streams” showed the lowest score variation, indicating the high operability of the item in cafés, while other items, i.e., “It is appropriate for green cafés to play audio recordings of the chirping sound of insects”, “It is appropriate for green cafés to play audio recordings of birds singing”, “It is appropriate for green cafés to play audio recordings of the calling sound of frogs”, and “It is appropriate for green cafés to play cellos music” showed high score variation. Although these items met the standard in terms of the average score, they did not attain an average score of 4 points or higher in the verification study with actual cafés, indicating that these items have low operability or would be impossible to implement.

#### 5.2.2. Visual Ambience Dimension

In this dimension, 3 items, i.e., “It is appropriate for green cafés to predominantly use white light”, “It is appropriate for green cafés to have flowers and plants”, and “It is appropriate for green cafés to plant flowers and trees outside the establishment”, showed high score variation and did not attain an average score of 4 points or higher in the verification study with actual cafés. Thus, these items have a low level of operability.

#### 5.2.3. Tactile Ambience Dimension

In this dimension, 7 items failed to meet the criterion in the verification study with actual cafés: “It is appropriate for green cafés to use electric fans for air circulation in the establishment”; “It is appropriate for green cafés to use air purifiers for fresh air in the establishment”; “It is appropriate for green cafés to use glass utensils, e.g., dinner trays, plates, knives, forks, and spoons”; “It is appropriate for green cafés to have windows to facilitate air circulation”; “It is appropriate for green cafés to use wooden wall facades”; “It is appropriate for green cafés to use stone tile floors”; and “It is appropriate for green cafés to use cement floors”. This finding indicates that these items have a low level of operability for implementation by cafés.

#### 5.2.4. Olfactory Ambience Dimension

In this dimension, only 1 item, “It is appropriate for green cafés to have a coffee aroma”, met the criterion in the verification study with actual cafés. Meanwhile, 3 items, i.e., “It is appropriate for green cafés to have a wood aroma”, “It is appropriate for green cafés to have a fruit aroma”, and “It is appropriate for green café stores to have a flower aroma”, did not meet the criterion of an average score of 4 points or higher and had large differences in their average scores, indicating that these items have a low level of operability for implementation by cafés.

#### 5.2.5. Taste Ambience Dimension

All 6 items in this dimension showed small differences in their average scores and the actual café verification results and had an average score of 4 points or higher, indicating that these items have a high level of operability for implementation by cafés.

## 6. Conclusions

After repeated feedback through three-round questionnaire surveys, among the 103 original indicators for the design of green café ambience, 36 indicators, including 5 indicators in the auditory ambience dimension, 7 indicators in the visual ambience dimension, 12 indicators in the tactile ambience dimension, 4 indicators in the olfactory ambience dimension and 8 indicators in the taste ambience dimension, were selected through stability analysis based on the criterion of an average importance score of 4 points or higher.

In this study, we found that because the concept of green café ambience has not yet been popularized, Taiwanese café operators would need to invest heavily in marketing to attract consumers based on ambience; therefore, it is recommended that when opening cafés, operators should take into account the capital and the regional target customer group. Based on the indicators selected by the experts in this study and the verification of the indicators with actual cafés, the items in the auditory ambience, tactile ambience, and olfactory ambience dimensions showed large variation in their scores; therefore, for the determination of green café ambience indicators in the future, items in these dimensions and the actual situation of café operations must be emphasized.

Further, when designing cafés, operators must consider the importance of ambience as experienced with all 5 senses; in the past, only visual ambience has been the focus of design [41,42], and the fact that humans receive information through 5 senses (touch, sight, hearing, smell, and taste) and assess this information with the brain to generate overall perception has not been considered [6,9,43]. The establishment of indicators of green café ambience design requires multi-party support and cooperation from academia, government, industry, and consumers, whose mutual assistance and joint efforts enable design implementation.

### Limitations and Future Research Directions

Among the indicators that were not included, 2 items, i.e., “It is appropriate for green cafés to have their service staff dress predominantly in green” in the visual ambience dimension and “It is appropriate for green cafés to have unique aromas of all types of food” in the olfactory ambience dimension, had importance scores of 4 points or higher. It is recommended that these items be included when new indicators are added in the future.

In this study, in the design of the green café ambience indicators, the “conceptual” principle, which is qualitative but not representative, was established set as the main research direction in the first stage. Therefore, it is recommended that investigators further formulate indicators for “specific manoeuvrability” to establish a complete café scale to provide a tool for the industry, governmental departments, and academia to evaluate green cafés in the future and to provide guidelines for green café practices.

In the present study, due to human power and time restrictions, cafés were not distinguished based on their size and regional characteristics in the discussion. There was also no analysis of consumers. Cafés in Taiwan are diverse; therefore, in the future, different types of cafés should be differentiated in terms of their size and category for in-depth examination. Green consumption is often associated with high prices, and economic factors affect people’s perceptions and quality of life, but this issue was not examined in this study. Therefore, it is recommended that in the future, price, income, and other economic factors be taken into account.

## Figures and Tables

**Table 1:** Proportions of experts from different fields.

Expert Category	Food and Beverage Business Management and Interior Design	Government Agencies	Café Operator
Number of experts	5	5	5

**Table 2:** Comparison of the screening results for the indicators using different criteria.

Screening Criterion	No. of Remaining Indicators	Remarks
Overall average score: 3.5 points	62	“Olfactory ambience” and “taste ambience” were eliminated
Importance score: greater than 4 points	40	All dimensions showed an important score of 4 or greater
Average score for each dimension	59	“Olfactory ambience” and “taste ambience” had scores lower than the overall average score, indicating uneven levels

**Table 3:** The operability of the indicators of green café ambience design.

Operativity	System Dimension	Selected Indicators	Average Importance Score		
			Scholar	Government	Business
	Auditory ambience	A1. It is appropriate for green cafés to play audio recordings of the sound of streams	4.6	4.5	4.7
		A2. It is appropriate for green cafés to play audio recordings of the chirping sound of insects	4.4	4.5	4.3
		A3. It is appropriate for green cafés to play audio recordings of the sound of birds singing	4.1	4.5	4.3
		A4. It is appropriate for green cafés to play cello music	4.1	4.5	4.2
		A5. It is appropriate for green café service staff to greet and converse with patrons in a predominantly soft tone	4.1	4.0	4.0
High	Visual ambience	V1. It is appropriate to decorate green cafés predominantly in wood tones	4.7	4.5	4.5
		V2. It is appropriate to decorate green cafés predominantly with transparent glass to introduce natural light into the establishment	4.4	4.5	4.7
		V3. It is appropriate for green cafés to use tables and chairs predominantly with warm colours	4.4	5.0	4.3
		V4. It is appropriate to decorate green cafés with flowers and plants	4.7	5.0	4.7
		V5. It is appropriate to plant flowers and trees outside green cafés	5.0	5.0	4.8
		V6. It is appropriate to light green cafés predominantly with warm colours	4.4	4.0	4.8
		V7. It is appropriate for green café service staff to dress predominantly in green and earth tones	4.4	5.0	4.3
	Tactile Ambience	T1. It is appropriate for green cafés to use electric fans for air circulation in the establishment	4.6	4.5	4.3
		T2. It is appropriate for green cafés to use dehumidifiers to dry the air in the establishment	4.1	4.5	4.2
		T3. It is appropriate for green cafés to use air purifiers to freshen the air in the establishment	4.3	4.5	4.3
		T4. It is appropriate for green cafés to use air conditioners to control the temperature in the establishment	4.1	4.0	4.3
		T5. It is appropriate for green cafés to have windows to facilitate air circulation	4.9	5.0	4.8
		T6. It is appropriate for green cafés to use wooden utensils, e.g., dinner trays, plates, knives, forks, and spoons	4.3	4.5	4.5
		T7. It is appropriate for green cafés to use glass utensils, e.g., dinner trays, plates, knives, forks, and spoons	3.9	4.0	4.3
		T8. It is appropriate for green cafés to use wooden wall facades	4.6	4.5	4.5
		T9. It is appropriate for green cafés to use wood floors	4.3	5.0	4.3
		T10. It is appropriate for green cafés to use stone tile floors	4.4	4.5	4.2
		T11. It is appropriate for green cafés to use seats made of wood	4.4	5.0	4.3
		T12. It is appropriate for green cafés to use tables made of wood	4.4	5.0	4.7
	Olfactory ambience	O1. It is appropriate for green cafés to have a coffee aroma	5.0	4.5	5.0
		O2. It is appropriate for green cafés to have a wood aroma	4.4	4.5	4.0
		O3. It is appropriate for green cafés to have a fruit aroma	4.4	4.5	4.2
		O4. It is appropriate for green cafés to have a food aroma	4.3	4.5	4.0
	Taste Ambience	t1. It is appropriate for green cafés to provide predominantly fresh vegetables	5.0	4.5	4.8
		t2. It is appropriate for green cafés to provide predominantly bland food	4.3	4.5	4.2
		t3. It is appropriate for green cafés to provide food predominantly in its original flavour	4.7	4.5	4.6
		t4. It is appropriate for green cafés to provide predominantly light food	4.6	4.0	4.8
		t5. It is appropriate for green cafés to provide juice drinks	4.1	4.0	4.4
		t6. It is appropriate for green cafés to provide dairy drinks	4.3	4.0	4.2
		t7. It is appropriate for green cafés to provide predominantly freshly made coffee drinks	4.0	4.0	4.8
		t8. It is appropriate for green cafés to provide predominantly freshly made juice drinks	4.1	4.5	4.5

**Table 4:** Selected indicators in this study for verification with actual cafés.

System Dimension	Indicator Content
Auditory ambience	A1. It is appropriate for green cafés to play audio recordings of the sound of streams
Visual ambience	V1. It is appropriate to decorate green cafés predominantly in wood tones
	V2. It is appropriate to decorate green cafés predominantly with transparent glass to introduce natural light into the establishment
	V3. It is appropriate for green cafés to use tables and chairs predominantly with warm colours
	V6. It is appropriate to light green cafés predominantly with warm colours
Tactile ambience	T4. It is appropriate for green cafés to use air conditioners to control the temperature in the establishment
	T6. It is appropriate for green cafés to use wooden utensils, e.g., dinner trays, plates, knives, forks, and spoons
	T9. It is appropriate for green cafés to use wood floors
	T11. It is appropriate for green cafés to use seats made of wood
	T12. It is appropriate for green cafés to use tables made of wood
Olfactory ambience	O1. It is appropriate for green cafés to have a coffee aroma
Taste ambience	t1. It is appropriate for green cafés to provide predominantly fresh vegetables
	t2. It is appropriate for green cafés to provide predominantly bland food
	t3. It is appropriate for green cafés to provide food predominantly in its original flavour
	t4. It is appropriate for green cafés to provide predominantly light food
	T7. It is appropriate for green cafés to provide predominantly freshly made coffee drinks
	T8. It is appropriate for green cafés to provide predominantly freshly made juice drinks
